# Ovarian Cancer: A Clinical Challenge That Needs Some Basic Answers

**DOI:** 10.1371/journal.pmed.1000025

**Published:** 2009-02-03

**Authors:** Kate Lawrenson, Simon A Gayther

## Abstract

Kate Lawrenson and Simon Gayther discuss two studies of ovarian cancer in*PLoS Medicine*, one on clinico-pathological heterogeneity and one on gene expression profiling.

From a clinical perspective, epithelial ovarian cancer is something of an enigma. Despite improvements in aggressive debulking surgery and the initial good response of patients to platinum-based chemotherapies, there has been little improvement in the survival rates for over three decades. About 65% of women with epithelial ovarian cancer will die within five years of their diagnosis [1]. Early-stage ovarian cancers are often asymptomatic and the recognised signs and symptoms, even of late-stage disease, are vague. Consequently, most patients are diagnosed with advanced disease, and it seems unlikely that symptoms alone could help to improve the proportion of tumours that are diagnosed at earlier, more treatable stages.

Unfortunately, there are no effective biomarkers that can identify early-stage disease and no reliable prognostic markers for predicting clinical response and guiding treatment regimes. Furthermore, there remains intense debate about the cellular origins, precursor lesions, and histological classification of the disease. With so many unknowns, it is perhaps not surprising that progress in reducing mortality in women diagnosed with ovarian cancer has been so limited.

## Two New Translational Research Studies

There is continued hope that the most recent scientific advances and discoveries will have the potential to affect patient care (translational research). For example, the last decade has seen revolutionary developments in the approaches used to characterise solid tumours at the molecular level. For some cancer types, the molecular characterisation of tumours has led to better strategies for predicting disease outcome, so that treatments can be targeted more effectively, and to the development of new therapies. Many of these approaches have been tried and tested for ovarian cancer too, but they have so far failed to deliver on the anticipation of a new biomarker or gene signature that can improve our understanding of the disease. Two studies published in *PLoS Medicine*, one in December 2008 and one in the current issue, shed some much needed light on the clinical challenge of ovarian cancer.

### Clinico-pathological heterogeneity in epithelial ovarian cancer.

The first study from Huntsman and colleagues [2] tackles the issue of clino-pathological heterogeneity in epithelial ovarian cancer. Scientists frequently refer to epithelial ovarian cancer as a single disease entity, even though it has been known for some time that this term describes a diverse group of tumours, each with different microscopic appearances and biological and genetic backgrounds. This diversity extends to clinical features of the disease; patients with different subtypes of ovarian cancer can respond differently to the same treatments and have different prognoses associated with their disease [3–5]. Arguably, the single feature that ovarian cancers have in common is the site of diagnosis.

**Linked Research Articles**

This Perspective discusses the following recent studies published in *PLoS Medicine:*

Crijns APG, Fehrmann RSN, de Jong S, Gerbens F, Meersma GJ, et al. (2009) Survival-related profile, pathways, and transcription factors in ovarian cancer. PLoS Med 6(2): e1000024. doi:10.1371/journal.pmed.1000024

Ate van der Zee and colleagues analyze the gene expression profiles of ovarian cancer samples from 157 patients, and identify an 86-gene expression profile that seems to predict overall survival.

Köbel M, Kalloger SE, Boyd N, McKinney S, Mehl E, et al. (2008) Ovarian carcinoma subtypes are different diseases: Implications for biomarker studies. PLoS Med 5(12): e232. doi:10.1371/journal.pmed.0050232

David Huntsman and colleagues describe the associations between biomarker expression patterns and survival in different ovarian cancer subtypes. They suggest that the management of ovarian cancer should reflect differences between these subtypes.

**Figure 1:**
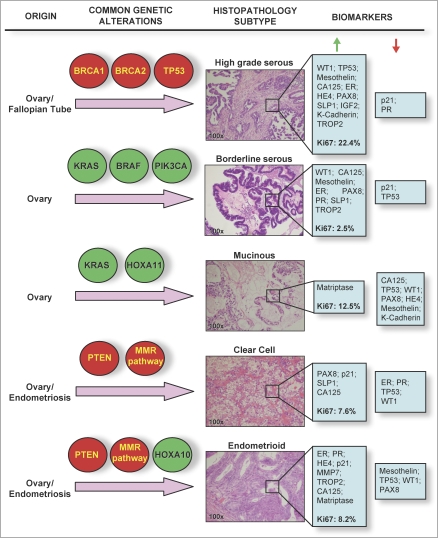
Histological and Molecular Heterogeneity in Epithelial Ovarian Cancers Common genetic alterations vary between different epithelial ovarian cancer sub-types. Highlighted in red are genes/pathways commonly inactivated in tumours; highlighted in green are genes commonly activated or amplified in epithelial ovarian cancer tumour specimens. Hematoxylin and eosin stained sections show typical histological and architectural appearance of the high-grade serous, borderline serous, mucinous, clear cell, and endometrioid sub-types. Biomarkers listed are those found in the study by Huntsman and colleagues to be highly expressed (i.e., samples positive in over 60% of tumours, green arrow), or lowly expressed (red arrow) in each histological sub-type. Median Ki67 labelling indices (a measure of the proportion of proliferating cells in a tumour sample) are given in bold type.

In order to define the different ovarian cancer sub-types at the molecular level, Huntsman and colleagues measured the expression of 21 candidate protein markers in 500 ovarian carcinomas representing the five main sub-types: high-grade serous, clear cell, endometrioid, mucinous, and low-grade serous. When the tumours were considered as a single phenotype, the investigators found ten biomarkers that were differentially expressed between early- and late-stage cancers. However, none of these markers varied with stage when this analysis was repeated after stratifying tumours by sub-type. Of the 21 different biomarkers tested, 20 differed significantly between sub-types, adding to a growing body of evidence showing that ovarian cancer heterogeneity is reflective of divergent molecular pathways underlying the development of the disease (Figure 1).

Several hypotheses have been suggested as a biological basis for this heterogeneity, such as the uncommitted state of the ovarian surface epithelium, and more recently, evidence that ovarian carcinomas can arise from a variety of precursor cell populations. For example, synchronous endometriosis is commonly observed in endometrioid and clear cell carcinomas, suggesting that at least one-third of these tumour types are associated with this benign lesion [6]. More recently, it has been suggested that at least a sub-set of epithelial ovarian cancers originate in the fallopian tube. Expression of the TP53 tumour suppressor protein in both ovarian inclusion cysts and the fallopian tube fimbriae (the distal portion of the fallopian tube) suggests that both could represent the origin of high-grade serous ovarian carcinomas [7,8]. An emerging hypothesis is that all ovarian cortical inclusion cysts that give rise to high-grade serous ovarian carcinomas originate from the fallopian tube; but this hypothesis will be a challenge to prove or disprove, since the hormone and growth-factor rich microenvironment of the ovarian stroma is likely to induce phenotypic changes in any epithelial cells that become trapped within it [8].

Huntsman and colleagues also looked at associations with outcome in their 21-biomarker panel. They found nine markers that were associated with differences in patient survival when the entire cohort was considered; but only three markers continued to show an association after sub-type stratification. To some degree, these data need to be treated with caution. Although this probably represents the most comprehensive immunohistochemistry based biomarker study to be published for ovarian cancer, after stratification into five sub-groups the number of cases within each sub-group limits the power of the study to detect significant associations and increases the likelihood of finding false positive associations. These findings will need validation in independent sample sets before researchers can feel confident that some of these prognostic markers might be of genuine use clinically.

The overarching message from Huntsman and colleagues' study is that biomarker expression is more strongly associated with histological sub-type than it is with disease stage. Looking to the future implications of these findings, a molecular profiling strategy may one day be a necessary diagnostic step in the clinical management of different ovarian cancer sub-types prior to making decisions on how to treat a patient's disease. A robust set of biomarkers in combination with universal guidelines for the classification of ovarian tumours will also be an essential feature of population-based studies of ovarian cancer that aim to identify any biomarker associated with any aspect of disease. There are additional impediments to the histological diagnosis of ovarian cancers that would benefit from such a molecular scoring system. For example, a proportion of ovarian cancers remain unclassified mainly because they are either undifferentiated or of mixed histology; and so biomarker profiling could provide an accurate method of differentiating tumours where the histopathological diagnoses are equivocal.

### Gene expression profiling of ovarian cancers.

The second study, by Crijns and colleagues [9], which is published in the current issue, takes a very different approach to the analysis of ovarian cancers (gene expression microarrays). Nevertheless, Crijns and colleagues' study echoes the underlying theme of Huntsman and colleagues' study in that the molecular analyses of tumours should be based on accurately defined histological sub-types of the disease. The use of gene expression profiling strategies to characterise human solid tumours was popularised after a study from van de Vijver and colleagues in 2002, which showed that gene expression signatures of breast cancers could be used to predict clinical outcome [10]. Since then, a similar approach has been used to characterise a multitude of different tumour types with varying degrees of success. For ovarian cancer, there are in excess of 40 published reports describing gene expression microarray analyses in ovarian tumours. However, a meta-analysis of 17 of these studies, which included 386 ovarian cancers, showed that there was very limited overlap between studies in the genes that were identified as “important” in ovarian cancer development [11].

This meta-analysis highlights many of the challenges of gene expression microarray studies and indicates why, most of the time, they have been unsuccessful in either identifying novel molecular targets of tumour development or gene signatures that can be used to predict clinical outcome. In a recent review, Tinker et al. neatly summed up many of these challenges [12]. The main issues relate to: the quality in the study design (e.g., using a well-defined phenotype that considers and adjusts for confounding factors); the quality of the experimental design (e.g., the microarray platform used and the tissues from which nucleic acids are extracted); and the statistical power of the study to detect meaningful effects after the analysis of several thousands of variables.

Crijns and colleagues' study is impressive in that it takes into account many of the potential pitfalls of gene expression microarray studies. As a result, it establishes a good chance of finding something meaningful. The main aim is clear and focused, asking the question, “Is there a genetic signature in advanced stage serous ovarian cancers that can predict survival after a diagnosis of the disease?” The study, which comprises 157 tumours, is substantially larger than most other studies of ovarian cancer that use a similar approach, but the selection of a specific tumour sub-type is perhaps the most significant strength in the study design. The authors identify a limited set of genetic events from the analysis of approximately 35,000 probes on a microarray, which provides a molecular signature that can distinguish between patients with good and bad prognosis. Using a cross validation approach that trained the data on 90% of the cases and tested the survival predictors on the remaining 10%, they were able to stratify patients into a low-risk group (mean survival time 41 month) and a high-risk group (mean survival time 19 months). This was statistically significant even after adjustment following permutation testing, suggesting it is unlikely that this result is due to over-fitting of the data.

## Next Steps

For studies such as those described by Huntsman and colleagues and Crijns and colleagues, there is a critical need to validate the findings in independent sample sets. This validation can be performed as part of an intra-study design, in which a second set of cases are evaluated by the same investigators using a similar experimental methodology; or, as Crijns and colleagues have done, by using publicly available data from one or more completely independent studies. Crijns and colleagues used published data from an expression microarray analysis of 118 primary serous ovarian cancers. Even though this analysis had been performed on a different microarray platform, the investigators were able to identify a 57-gene signature, which was a sub-set of their 86-gene signature, that was able to predict which patients fell into the low- and high-risk prognostic groups they had defined.

The findings of both of the studies described in this Perspective are very encouraging, given previous attempts to identify prognostic markers or molecular signatures for ovarian cancer. Much more extensive follow-up is now needed, requiring multi-centre collaborations and agreement on the most appropriate study designs, which will need to consider the challenges surrounding experimental methodology, quality control, statistical power, and (of course) phenotypic heterogeneity. As is so often the case, breast cancer research in this area is at a much more advanced stage and represents something of a paradigm. There is now good evidence that gene expression signatures that predict prognosis in breast cancer, generated from a multitude of independent studies, can be validated in multi-centre studies [13,14]. This has led to the initiation of two prospective randomised clinical trials to test the efficacy of using expression profiling in clinical practice [15].

Could these molecular tools ultimately be used to guide personalised treatment for patients with ovarian cancer? The work of Huntsman and Crijns and colleagues, and the example of breast cancer, certainly suggests that this may be feasible—but only if ovarian cancers are appropriately classified. The first step would seem to be to get an agreement amongst researchers and clinicians about what this classification comprises. Only then does it seem likely that inroads can be made into the mortality statistics that so consistently define this disease.
